# Cep126 is required for pericentriolar satellite localisation to the centrosome and for primary cilium formation

**DOI:** 10.1111/boc.201300087

**Published:** 2014-07-09

**Authors:** Raffaella Bonavita, Dawid Walas, Anna K Brown, Alberto Luini, David J Stephens, Antonino Colanzi

**Affiliations:** * Institute of Protein Biochemistry, National Research CouncilNaples, 80131, Italy; † Telethon Institute of Genetics and Medicine (TIGEM)Naples, 80131, Italy; ‡ Cell Biology Laboratories, School of Biochemistry, Medical Sciences Building, University of BristolBristol, BS8 1TD, UK

**Keywords:** Centrosome, Cep126, Microtubules Primary Cilium, Pericentriolar Satellites

## Abstract

**Background Information:**

The centrosome is the primary microtubule-organising centre of animal cells and it has crucial roles in several fundamental cellular functions, including cell division, cell polarity, and intracellular transport. The mechanisms responsible for this are not completely understood.

**Results:**

The poorly characterised protein CEP126 localises to the centrosome, pericentriolar satellites and the base of the primary cilium. Suppression of CEP126 expression results in dispersion of the pericentriolar satellites and disruption of the radial organisation of the microtubules, and induces disorganisation of the mitotic spindle. Moreover, CEP126 depletion or the transfection of a CEP126 truncation mutant in hTERT-RPE-1 and IMCD3 cells impairs the formation of the primary cilium.

**Conclusions:**

We propose that CEP126 is a regulator of microtubule organisation at the centrosome that acts through modulation of the transport of pericentriolar satellites, and consequently, of the organisation of cell structure.

## Introduction

The centrosome functions as the primary microtubule (MT)-organising centre. The centrosome is composed of two barrel-shaped centrioles that are held within a proteinaceous matrix of pericentriolar material. This pericentriolar material and the centrosome regulate several distinct processes, including MT nucleation, anchorage and release (Nigg and Raff, 2009). MTs are nucleated from γ-tubulin ring complexes (Moritz and Agard, 2001). The ‘pericentriolar satellites’ have a fundamental role in the regulation of the centrosomal anchoring function. These are granular structures that surround the centrosome and have been implicated in the regulation of the assembly and targeting of protein complexes to the centrosome, which is essential for the anchoring of MTs (Kubo et al., 1999).

The centrosome is essential to many cellular functions, as the MT network is an intrinsic part of cell polarity, motility and division (Sutterlin and Colanzi, 2010). In actively dividing cells, the centrioles of each centrosome are duplicated to organise the poles of the MT-based mitotic spindle that are essential for correct segregation of the genetic material (Nigg and Stearns, 2011). In most quiescent non-dividing epithelial cells, the centrosome migrates to the apical cell surface, where the mother centriole docks with the plasma membrane and forms the basal body. This leads to the generation of the primary cilium, which is a specialised projection of the plasma membrane (Sorokin, 1962; Singla and Reiter, 2006) that has crucial roles in the regulation of developmental pathways and tissue homeostasis (Veland et al., 2009). Defects in genes associated with cilium assembly and function are linked to a wide range of developmental disorders and diseases that are collectively known as ciliopathies (Waters and Beales, 2011). Ciliopathies can result in complex, multisystem pathologies, and they are associated with low quality of life and early death of many patients (Tobin and Beales, 2009).

Unfortunately, many crucial aspects of centrosome function remain unclear, including the basic molecular mechanisms that underlie the mechanisms of MT nucleation, anchoring and stabilisation and the regulation of centrosome positioning within the cell. The centrosome is composed of hundreds of proteins, many of which have not yet been characterised (Andersen et al., 2003). Here, we show that the poorly characterised protein Cep126 (for centrosomal *p*rotein of ∼126 kDa), previously known as KIAA1377, (Chen et al., 2009; Lim et al., 2012; Tipton et al., 2012) is a novel and fundamental regulator of centrosomal function. Cep126 localises to the centrosome and pericentriolar satellites, and it is necessary for MT organisation and the association of the MT array with the centrosome. We also show that Cep126 is required for the formation of the primary cilium.

## Results

### Dynamic localisation of Cep126 to the centrosome

**Figure 1:**
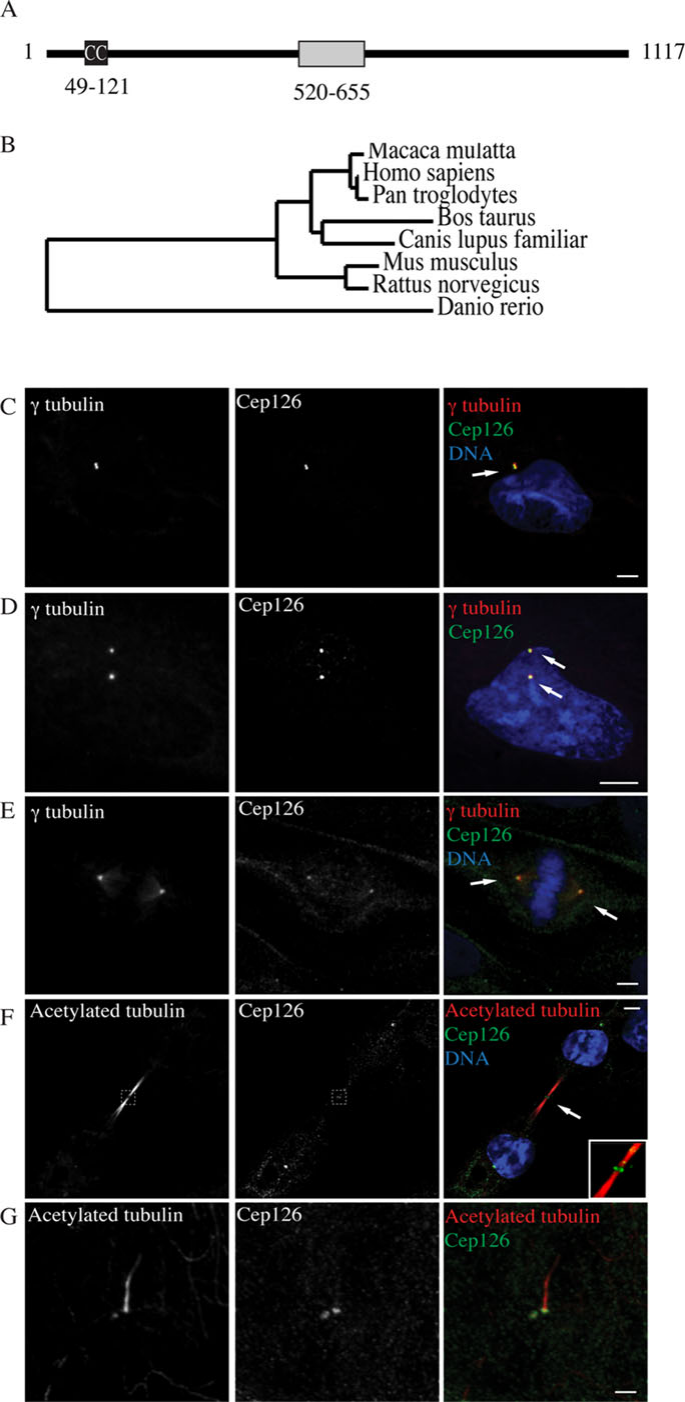
Cep126 localises to the centrosome (A) A diagram showing putative structural domains of Cep126 identified by sequence analysis using Swiss-Prot and SBASE databases (www.uniprot.org/uniprot/Q9P2H0; http://hydra.icgeb.trieste.it/sbase). A coiled-coil domain (black box, CC) and a putative centrosomal localisation domain (grey box) are present in Cep126. (B) A phylogenetic tree of Cep126 homologs. (C–G) hTERT-RPE-1 cells were grown on glass coverslips, fixed with methanol for 5 min at −20°C and treated for immunofluorescence with an anti-Cep126 antibody (green), and an anti-γ-tubulin antibody (red) or an anti-acetylated-tubulin antibody (red). Representative confocal microscopy images show that Cep126 localises mainly at the centrosome in cells in interphase (C), G2 (D) and mitosis (E). Cep126 localises also to the midbody during cytokinesis (F). (G) hTERT-RPE-1 cells were grown on glass coverslips, serum starved for 48 h, fixed, and stained with an anti-Cep126 antibody (green) and an anti-acetylated tubulin antibody (red) to indicate the cilium axoneme. Scale bars: 4 µm.

Cep126 is a poorly characterised protein. Previous studies based on Cep126 transfection have indicated that it is mainly localised at the centrosome and the midbody (Chen et al., 2009; Tipton et al., 2012). Sequence analysis of Cep126 did not show significant homology with proteins of known function, but revealed the presence of a coiled-coil domain (region 49–121) and of a putative centrosome-localisation region (region 520–655) (Figure1A). Phylogenetic analysis indicated that homologs of Cep126 are present in most vertebrates (Figure1B), whereas there are no homologues in invertebrates such as *Drosophila melangaster* or *Caenorhabditis elegans*.

To investigate the role of Cep126, we first studied the localisation of the endogenous protein using confocal microscopy. To this purpose, human telomerase immortalised retinal pigmented epithelial (hTERT-RPE-1) cells were stained with an anti-Cep126 antibody and an anti-γ-tubulin antibody, to identify the centrosome. Confocal microscopy showed that in both interphase and mitosis, Cep126 localises mainly at the centrosome (Figures1C–1E, arrows) and showed also a minor component of faint cytoplasmic dots (Figures1C–1E). During cytokinesis, Cep126 is also associated with the midbody (Figure1F), in agreement with the existing data based on Cep126 overexpression (Chen et al., 2009; Tipton et al., 2012). The cells were also serum-starved (a condition that promotes cilium formation), fixed and stained with an anti-acetylated-tubulin antibody, to identify the MT axoneme within the cilium. In these hTERT-RPE-1 cells, Cep126 localises mainly at the base of the primary cilium (Figure1G).

**Figure 2:**
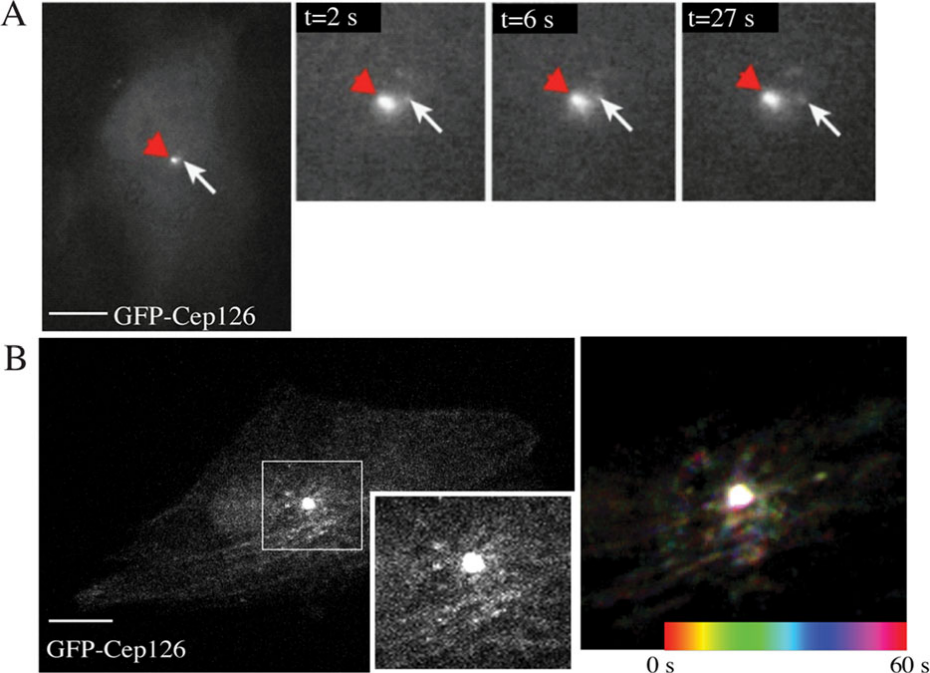
Dynamic localisation of Cep126 The dynamics of Cep126 localisation were investigated using live-cell imaging. hTERT-RPE-1 cells were transfected with Cep126-GFP. As indicated by arrows in (A), Cep126 was associated not only with the centrosomes, but also with the pericentrosomal material, and showed limited movements around the centrosome, both towards and away from the centrosome. (B) Pseudocolouring of the time points shows a dynamic population that undergoes local short-range translocation, presumably along MTs. Scale bars: 10 µm.

Next, the dynamics of Cep126 localisation were investigated using live-cell imaging. This analysis showed that Cep126-GFP is associated not only to centrosomes, but also to particles that could represent pericentrosomal material (Figures2A and 2B, and Supplementary Movie). This localisation is not evident by immunolabelling of the endogenous Cep126 protein in fixed cells. This might be due to non-accessibility of the antigen, to the sensitivity of the available antibodies, or to experimental conditions (*e.g*., a differential level of abundance of Cep126 between centrosome and satellites). This pericentrosomal pool of Cep126 showed limited movements around the centrosome (Figure2A, arrows; Supplementary Movie). Further analysis revealed that the small dynamic spots of Cep126 showed bidirectional motility, so both towards and away from the centrosome (Figure2A, arrows; Supplementary Movie). Pseudocolouring over time revealed a dynamic population of Cep126 that undergoes local short-range translocation (Figure2B), which appears to occur along MTs, as was shown for the pericentrosomal component PCM1 (Kubo et al., 1999).

**Figure 3:**
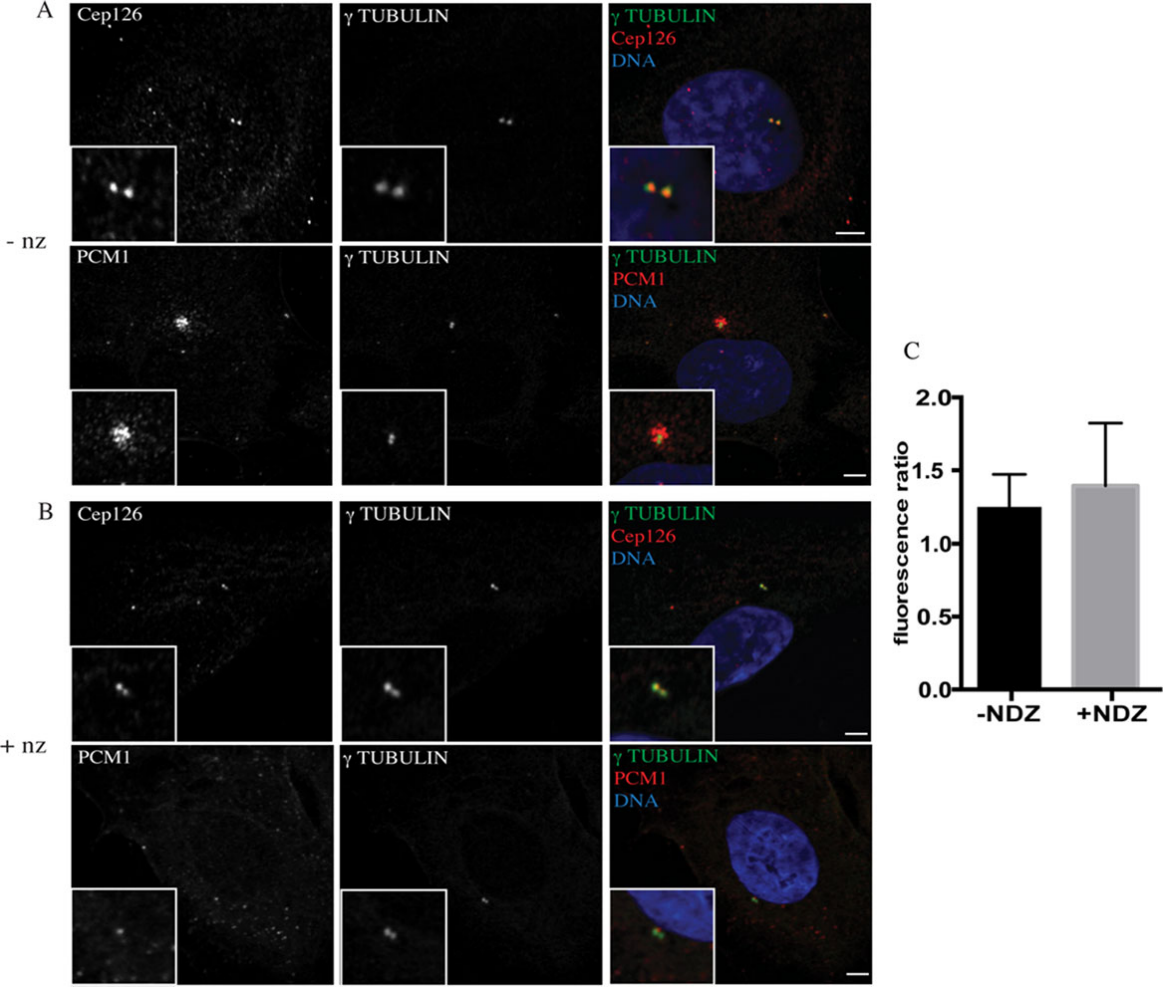
Cep126 localised to the centrosome in a MT-independent manner hTERT-RPE-1 cells were untreated (A) or treated with 33 µg/ml NZ for 3 h (B), and stained with an anti-α-tubulin antibody (red) and with anti-Cep126 or anti-PCM1 antibodies (green). Representative images showing that after NZ treatment, Cep126 retained its centrosomal localisation (A), whereas PCM1 showed a dispersed distribution (B). (C) Quantification of Cep126/γ-tubulin fluorescence ratio in not treated (black bar, −NZ) and NZ-treated (grey bar, +NZ) cells; no statistically significant differences have been observed. Data in (C) are means ± SD of three independent experiments; more than 15 cells where counted per experimental condition.

We then analysed the localisation pattern of Cep126 and PCM1 in hTERT-RPE-1 cells following depolymerisation of the MT network with nocodazole (NZ). When these cells were treated with NZ, the pericentrosomal and granular staining pool of PCM1 was completely dispersed, as would be expected for proteins associated with pericentriolar satellites. In addition, although PCM1 was completely dissociated from the centrosome, Cep126 retained its centrosomal localisation (Figures3A and 3B), as shown by measuring the fluorescence ratio between Cep126 and γ-tubulin in control and NZ-treated cells (Figure3C). Moreover, disruption of pericentriolar satellites transport by blocking the dynein–dynactin complex through the overexpression of p50 dynamitin caused dissociation of PCM1 from the centrosome, whereas Cep126 localisation was not affected (Supplementary Figure S1, arrows). Collectively, these data indicate that Cep126 is localised to the centrosome in a MT-independent manner. This has also been reported for other pericentriolar satellites and for centrosomal proteins such as FOR20, OFD1 and pericentrin, which have two pools, one specifically on the centrosome, and the other on pericentriolar satellites (Gillingham and Munro, 2000; Sedjai et al., 2010; Lopes et al., 2011).

Next, we analysed the dynamics of the centrosomal pool of Cep126-GFP using fluorescence recovery after photobleaching (Supplementary Figures S2A and S2B). This analysis showed that the half-life of Cep126 association was 40.7 ± 12.8 s, and also revealed that most of the Cep126 was immobile (66.9 ± 9.2%). This finding suggests that there are two distinct populations of Cep126 at the centrioles: one pool that is dynamic, and another that is stably associated with the centrioles.

### Cep126 is required for pericentriolar-satellite localisation to the centrosome and for MT organisation

**Figure 4:**
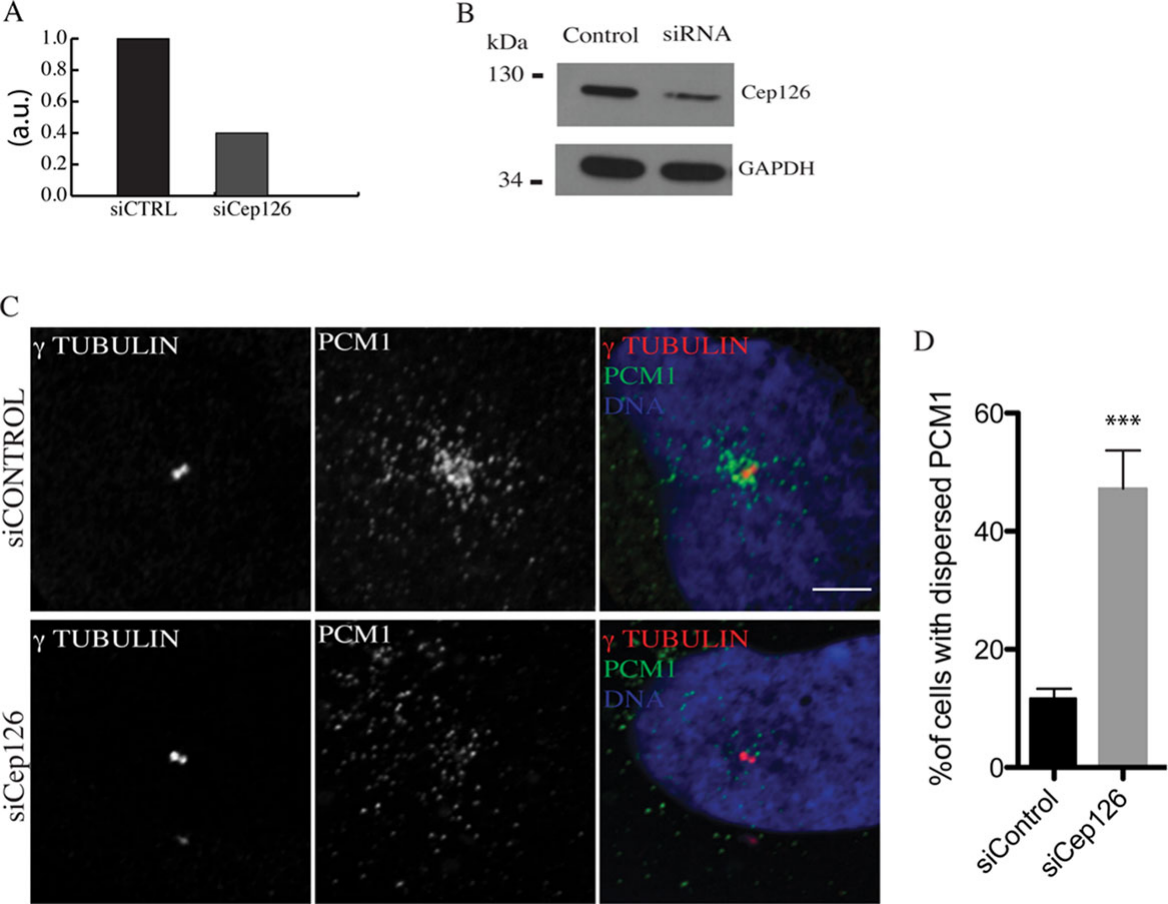
Cep126 depletion affected pericentriolar-satellite localisation hTERT-RPE-1 cells were transfected with non-targeting and Cep126-targeting siRNAs for 48 h, with representative images shown. (A) Depletion of Cep126 was monitored by RT-PCR. (B) Depletion of Cep126 was monitored by immunoblotting. (C and D) Cep126 depletion leads to dispersal of PCM1. The cells were fixed in methanol and stained with an anti-γ-tubulin antibody (red) as marker of the centrosome, and with an anti-PCM1 antibody (green) to stain the pericentriolar satellites. (D) Quantification of PCM1 dispersion in cells treated with non-targeting and Cep126-targeting siRNAs. Data in (D) are means ± SD of three independent experiments; more than 200 cells were counted per experimental condition. *** indicate *P* < 0.0001, using *t*-test. Scale bars: 4 µm.

**Figure 5:**
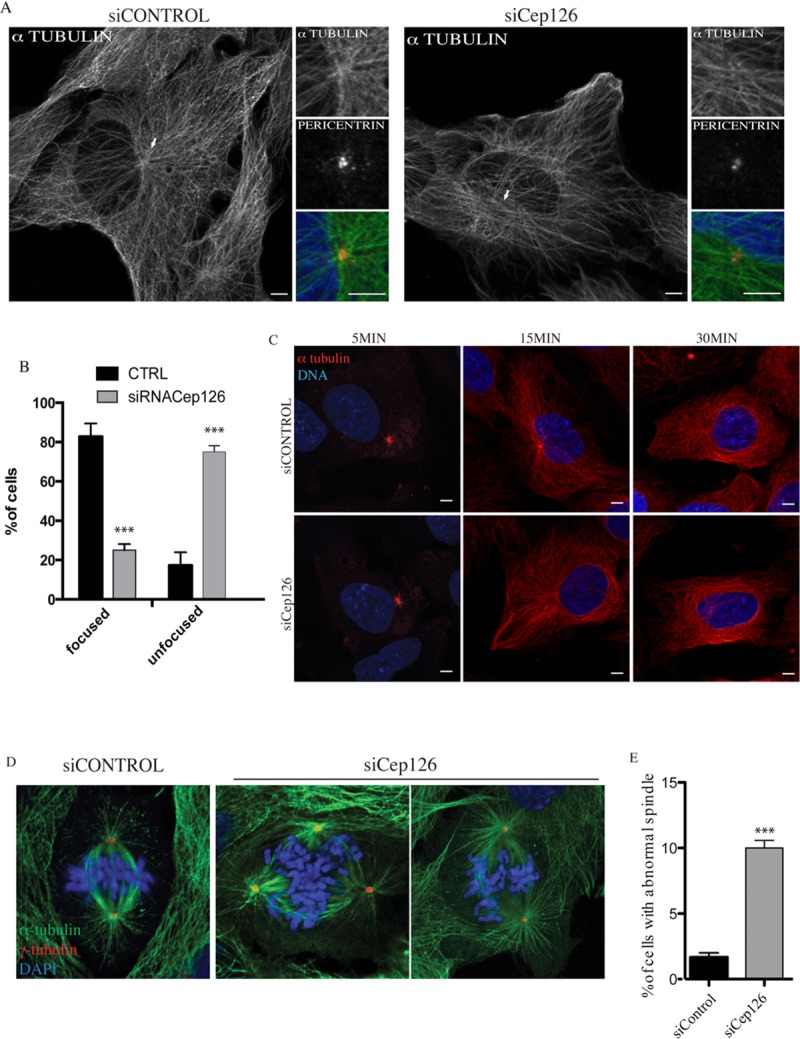
Cep126 depletion affected microtubule (MT) organisation hTERT-RPE-1 cells were transfected with non-targeting (siCONTROL) and Cep126-targeting siRNAs (siCEP126) for 48 h, with representative images shown. (A) hTERT-RPE-1 cells were fixed in 4% paraformaldehyde and stained with an anti-pericentrin antibody (red) as marker of the centrosome, and with an anti-α-tubulin antibody (green) as marker of the MT cytoskeleton; Cep126 depletion affected MT organisation as indicated by the arrows. (B) Quantification of the MT organisation phenotypes. (C) Cep126 is not involved in MT nucleation. The cells were treated with NZ and fixed at different times after NZ wash-out. An anti-α tubulin antibody (red) was used as marker of MTs. (D and E) Cep126 depletion induces an alteration of the mitotic spindle in mitotic cells. hTERT-RPE-1 cells were transfected with control siRNA or with siRNA against Cep126 for 48 h. (D) Cells were fixed and stained with antibodies against γ-tubulin (red) and α-tubulin (green) to visualise the mitotic spindle. (E) Quantification of the mitotic defects observed in control and Cep126 depleted cells. Data in (B and E) are means ± SD of three independent experiments; more than 200 cells were counted per experimental condition. *** indicates *P* < 0.0001, using *t*-test. Scale bars: 4 µm.

Given Cep126 localisation, we addressed the role of Cep126 in a series of centrosome-based functions, including the organisation of MTs and the localisation of pericentriolar satellites (Barenz et al., 2011). hTERT-RPE-1 cells were treated with a control small-interfering (si)RNA and siRNAs against Cep126 for 48 h. Then, the cells were fixed and analysed for immunofluorescence. This Cep126 silencing was confirmed by RT-PCR (Figure4A), Western blotting (Figure4B) and immunofluorescence (Supplementary Figure S3). Then, the effect of Cep126 depletion was monitored through the pericentriolar-satellite component PCM1 (Barenz et al., 2011). This analysis revealed that while control cells showed that the pericentriolar satellites were closely associated with the centrosome in 90% of the cells, in the Cep126-depleted cells, the pericentriolar satellites were dispersed and located away from the centrosome in about 50% of the cells (Figures4C and 4D), indicating that Cep126 depletion severely affected pericentriolar-satellite localisation. In addition, we investigated the organisation of MTs in these Cep126-depleted cells by examining projections of six confocal ‘slices’ that spanned ±0.4 µm up and down a focal plane focused on the centrosome. Our analysis showed a subtle but functionally important difference in the overall organisation of the MT fibres. In control cells, the MTs formed a radial array that emerged from the centrosome and extended towards the cell periphery (Figure5A, left panel, arrow). Conversely, in Cep126-depleted cells, the MTs did not radiate from the centrosome, with most of the MTs randomly oriented throughout the cytoplasm (Figure5A, right panel, arrow). Although a focused array was present in more than 80% of control cells, less than 20% of Cep126 knock down cells showed a focused array (Figure5B). Moreover, the localisation of pericentrin was also altered in the Cep126-depleted cells. Indeed, although the centrosomal pool of pericentrin was only slightly decreased (Supplementary Figure S4), the pericentriolar-satellite-associated pool seen in control cells (Figure5A, siCONTROL) was completely dispersed after Cep126 knockdown (Figure5A, siCEP126). This is in agreement with the concept that the fraction of pericentrin that is associated with pericentriolar satellites requires an intact and centrosome-focused array of MTs or the correct transport of pericentriolar satellites along MTs, whereas the centrosomal pool is recruited through the PACT (pericentrin-Akap450 centrosomal targeting) domain in a MT-independent manner (Gillingham and Munro, 2000). This phenotype is consistent with either defective MT growth from the centrosome or inefficient anchoring of MTs to the centrosome (Delgehyr et al., 2005).

Thus, we then determined whether this Cep126 depletion affects the MT nucleation process. To depolymerise the MTs, the control and Cep126-depleted hTERT-RPE-1 cells were treated with 33 µM NZ for 2 h at 37°C, and then incubated for 40 min at 4°C, and the MT nucleation was analysed at different times following NZ wash-out and incubation at 37°C. Similar to control cells, the MT aster was visible 5 min after NZ wash-out and MTs continued to grow from the centrosome, becoming more evident after 30 min of incubation in fresh media (Figure5C). Importantly, there were no differences in the re-formation rates of the MT aster between the control and Cep126-depleted cells at 5 and 15 min of observation. However, after 30 min of incubation in fresh medium, a fraction of the Cep126-depleted cells showed MT fibres that were not focused on the centrosome (Figure5C), in agreement with the effects on MT organisation seen previously in Cep126-depleted cells (see Figure5A). Collectively, these data show that Cep126 is not involved in the initiation or polymerisation of MTs, but is instead implicated in the transport of pericentriolar satellites to the centrosome, and as a consequence, in the regulation of the anchoring of newly formed MTs to the centrosome (Figures5A and 5C) (Kim et al., 2004; Delgehyr et al., 2005; Barenz et al., 2011). As further evidence of an essential role of Cep126 in MT organisation, staining with an antibody to α-tubulin of mitotic cells showed that while control cells contained predominantly bipolar spindles that aligned the DNA in the metaphase plate, multiple disorganised spindle asters were observed in Cep126-depleted cells (Figures5D and 5E) (Kim and Rhee, 2011).

**Figure 6:**
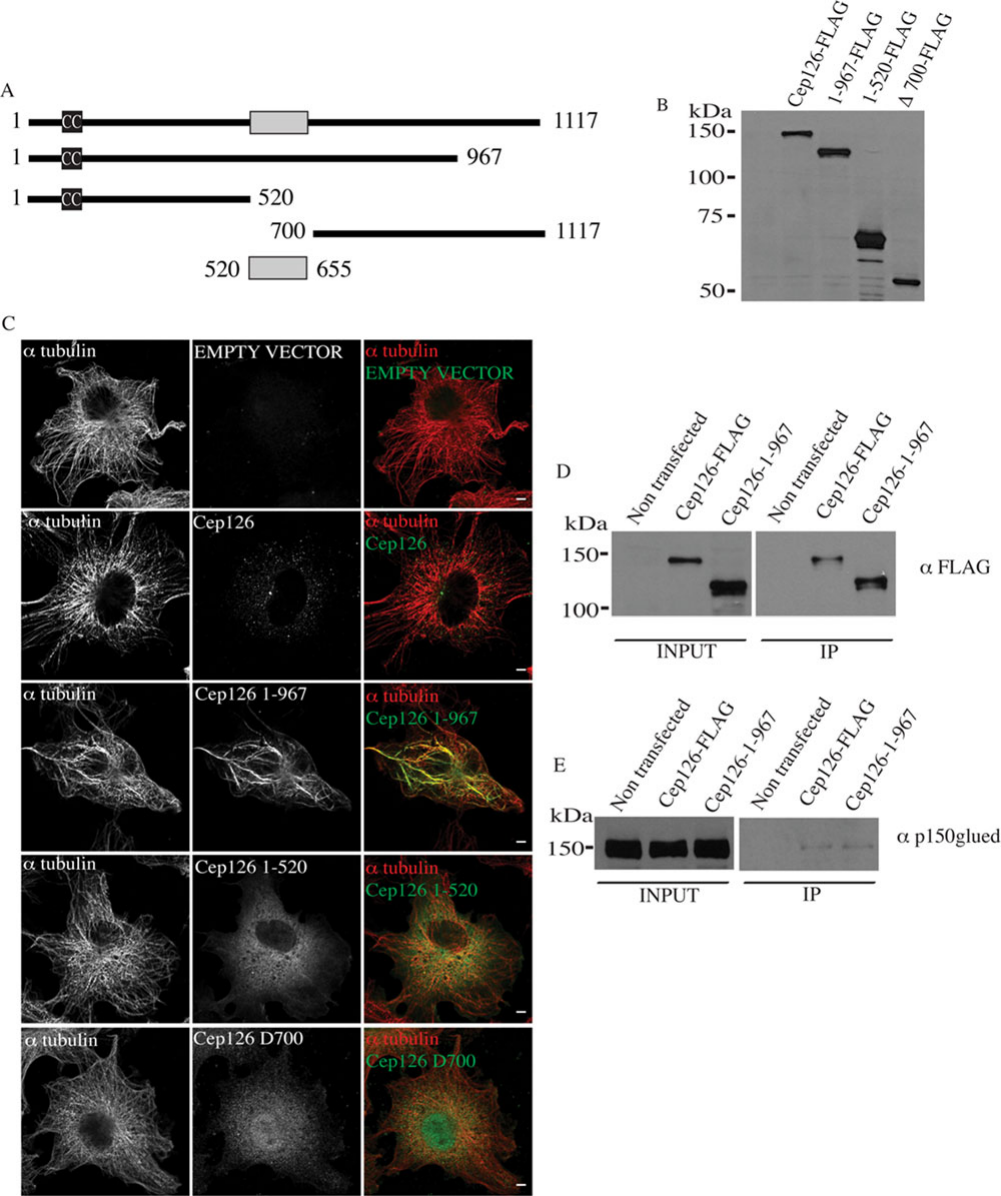
Cep126 interacts with p150Glued (A–C) Cep126 deletion mutants affect MT anchoring to the centrosome. (A) Schematic representation of Flag-tagged full-length Cep126 and the four truncation mutants. (B) Representative Western blot (using an antibody against Flag) of hTERT-RPE-1 cell extract from cells expressing the full-length Cep126-Flag and the indicated truncation mutants, showing that the proteins produced are of the predicted sizes. (C) The 1–967 Cep126, (but not 1–520 and Δ700 Cep126) affects MT cytoskeleton organisation. Representative images of hTERT-RPE-1 cells transfected with full length Cep126-FLAG and the four mutants. The cells were fixed with 4% paraformaldehyde and stained with an anti-Flag antibody (green) to label the transfected cells, and with an anti-α-tubulin antibody (red) for the MTs. (D and E) Cep126 full-length and the 1–967 truncation mutant interact with p150Glued. (D) Representative Western blot of hTERT-RPE-1 cell extracts of not transfected and expressing the full-length Cep126-Flag or the 1–967 Cep126-Flag truncation mutant, using an anti-Flag antibody, shows that these proteins are expressed and immunoprecipitated with the anti-Flag antibody. (E) Representative Western blot using an anti-p150Glued antibody shows that both of these forms of Cep126 interact with p150Glued. Scale bars: 4 µm.

To investigate further the function of Cep126 in MT anchoring, and to circumvent possible problems of interpretation due to the effects of Cep126 on the mitotic spindle, we followed the approach of the construction of a series of Cep126 deletion mutants and test their effects at short transfection times. These were then investigated in COS7 cells, as these cells have a well-defined astral MT array that radiates out from the centrosome, and thus they represent a suitable model for investigating alterations in MT organisation (Kim et al., 2004). Thus, we constructed Flag-tagged Cep126-deletion mutants that express the 1–520 and 700–1117 (Δ700) variants of Cep126 (upstream and downstream of the putative centrosomal-localisation region, see Figure1B), a mutant that contains the putative centrosome-localisation region but lacks the C-terminal domain (968–1117), and a mutant that contains the only the putative centrosome-localisation region (520–655) (Figures6A and 6B).

As expected, Cep126-Flag localised to the centrosome (Supplementary Figure S5), and on MTs at high expression levels (Tipton et al., 2012). The cells transfected with the control vector or with Cep126-Flag did not show any detectable alterations in their MT structure after 20 h of expression (Figure6C). However, the mutant lacking the C-terminal domain was still localised at the centrosome (Supplementary Figure S5; Figure6C), although its expression resulted in severe impairment of MT organisation, whereby the MT fibres lost their radial orientation, and were instead organised in bundles (Figure6C). Overexpression of the mutant lacking the C-terminal domain also resulted in the dispersion of the pericentriolar satellites (not shown), similarly to what was shown for the Cep126-depleted cells (Figures4C and 4D). In contrast, the 1–520 and Δ700 Cep126 variants, which do not contain the predicted centrosomal-localisation domain, did not localise to the centrosome and did not show detectable effects on MT organisation (Figure6C). Finally, the mutant expressing the predicted centrosome-binding domain formed protein aggregates (Supplementary Figure S6) and thus its function was not investigated. Thus, although our findings are in agreement with an important role of the 520–655 region in regulating centrosome localisation, it is plausible that this region is not structurally stable and its exact role remains to be determined. Collectively, these observations complement and confirm the finding that MTs and pericentriolar satellites (Figures4C and 4D) are dissociated from the centrosome when Cep126 function is affected (Figures5A and 6C).

### Cep126 interacts with p150Glued

We find that Cep126 depletion caused dispersion of PCM1 and pericentrin away from the centrosome (Figures4C and 4D; Supplementary Figure S4). PCM1 and pericentrin are maintained on pericentriolar satellites via a mechanism that involves the dynein–dynactin complex, as this complex is necessary for transport to the centrosome of factors that are required for MT anchoring to the centrosome (Quintyne et al., 1999). Thus, we investigated whether Cep126 is part of a complex that contains p150Glued, an important subunit of the dynein–dynactin complex. hTERT-RPE-1 cells were transiently transfected with full-length Cep126-Flag and the 1–967 Cep126 deletion mutant. Immunoprecipitation of these Cep126 constructs indicated that both of these proteins interact with p150Glued (Figures6D and 6E). Moreover, and in support of the hypothesis that Cep126 regulates dynein/dynactin-mediated transport of pericentriolar satellites, expression of the 1–967 Cep126 deletion mutant reduced the centrosome levels of p150 (Supplementary Figure S7), whereas Cep126 depletion and overexpression of the 1–967 Cep126 deletion mutant resulted in the fragmentation and dispersion of the Golgi complex (Supplementary Figure S8).

### Cep126 is required for cilium formation

**Figure 7:**
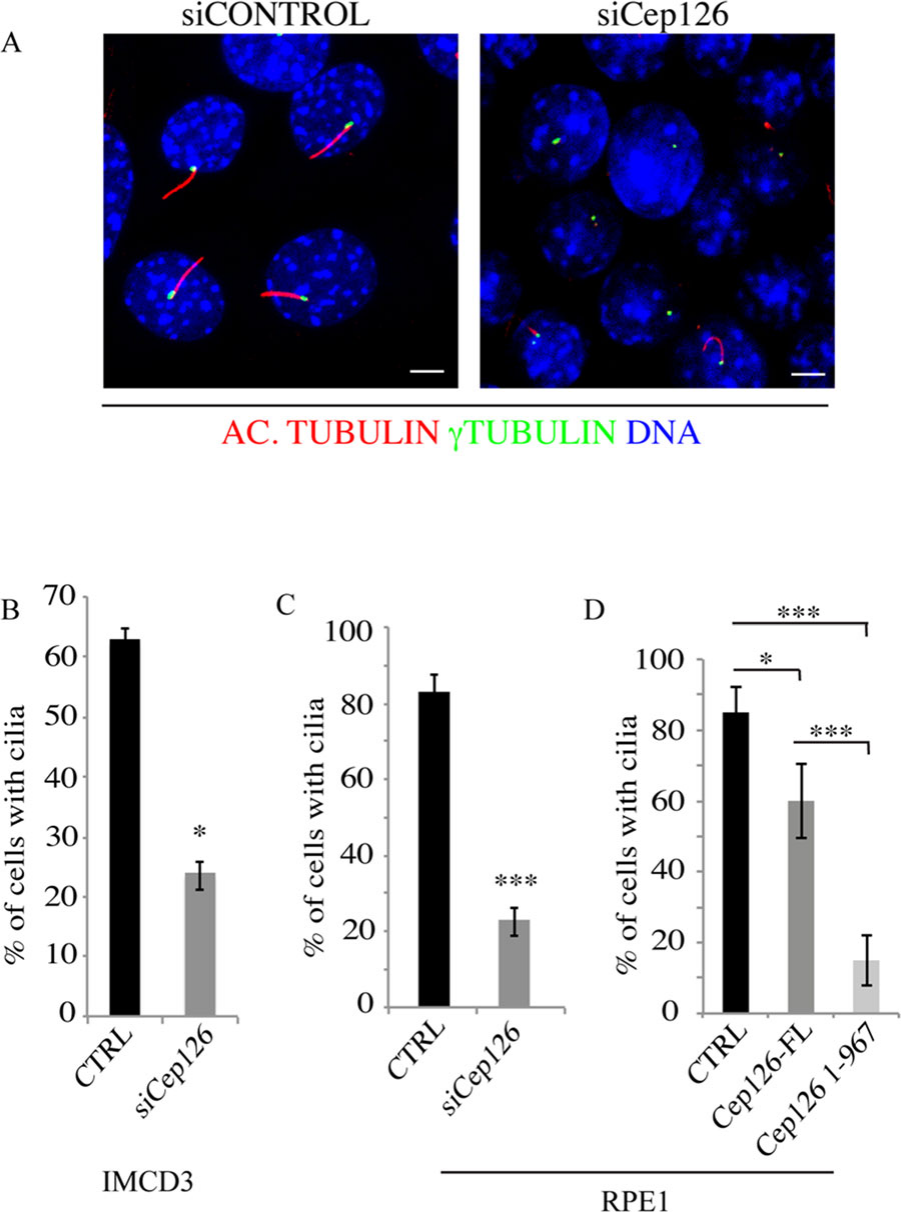
Cep126 is involved in cilium formation (A) Representative images of IMDC3 cells transfected with non-targeting (siCONTROL) and anti-Cep126 siRNAs (siCEP126), and incubated for 48 h in the absence of serum. The cells were fixed in methanol and stained with an anti-acetylated tubulin antibody (red) and an anti-γ-tubulin antibody (green). The presence of a detectable cilium was monitored by confocal microscopy. (B and C) Quantification of the cells treated as in (A) for the percentages of IMCD3 (B) and hTERT-RPE-1 (C) cells showing a detectable cilium; the reduction of cilium formation was statistically significant. (D) hTERT-RPE-1 cells were transfected with an empty vector as the control (CTRL), and full-length Cep126 (Cep126-FL) and 1–967 Cep126 depletion mutant (Cep126 1–967). The cells were serum starved for 48 h, and fixed and treated for immunofluorescence to monitor cilium formation. Data in (B–D) are means ± SD of three independent experiments; more than 200 cells where counted per experimental condition. Scale bars: 4 µm. *P < 0.001, ***P < 0.0001, using *t*-test.

Given the localisation of Cep126 to the centrosome and the basal body of the primary cilium, we investigated whether Cep126 depletion causes defects in cilium formation. For this, we used IMCD3 and hTERT-RPE-1 cells, as these cells are well-characterised *in vitro* models to study the formation of the primary cilium. IMDC3 and hTERT-RPE-1 cells were seeded on glass coverslips and transfected for 48 h in the absence of serum with two different sets of anti-Cep126 siRNAs, to reduce the possibility of off-target effects. The cells were then stained with an anti-acetylated-tubulin antibody to identify the cilium, and the cells that showed a fully formed cilium were counted (Figure7A). In line with an essential role of Cep126 in cilium formation, the percentage of ciliated cells seen in the Cep126-depleted cells was strongly decreased compared with that seen in the control-treated cells (Figures7B and 7C).

To address the role of Cep126 by a different approach, the cells were serum-starved and transfected with the 1–967 Cep126 truncation mutant, which severely impaired MT organisation (Figure6C). The hTERT-RPE-1 cells where transfected with the empty vector (control) and vectors that expressed full-length Cep126-Flag and the Cep126(1–967)-Flag truncation mutant. The expression of the 1–967 Cep126 truncation mutant caused more than 80% reduction in ciliated cells (Figure7D). In comparison, the cells transfected with full-length Cep126 showed a modest, but significant, reduction when compared with control non-transfected cells (Figure7D), which appeared to be a consequence of the high level of expression seen in a fraction of the cells.

In conclusion, these data demonstrate that Cep126 has an essential role in cilium formation.

## Discussion

In this study, we describe the first functional characterisation of Cep126. We have shown that Cep126 localises to the centrosome throughout the cell cycle. In addition, live imaging of cells with overexpressed Cep126 reveal its localisation in pericentriolar satellites, which are small and dynamic granular structures that are involved in the transport of proteins that regulate a large series of crucial centrosome functions (Barenz et al., 2011). The localisation of Cep126 with pericentriolar satellites is not evident by immunolabelling of the endogenous Cep126 protein in fixed cells. Moreover, our study has revealed that Cep126 physically interacts with p150Glued and is required for centrosomal accumulation of pericentriolar satellites. The functional relevance of Cep126 is highlighted by our demonstration that depletion of Cep126 strongly impairs radial MT organisation, spindle organisation and primary cilium formation.

Based on these data, we propose that Cep126 is an essential regulator of pericentriolar-satellite transport to the centrosome, and is thus crucial for centrosomal recruitment of proteins involved in a series of centrosome-based functions. Indeed, these centrosome-based functions are probably not limited to the spindle and cilium assembly defects described in the present study, but potentially include cell polarisation and migration.

One of the basic defects observed in Cep126-depleted cells is the lack of a centrosome-focused MT array. The stabilisation and anchoring of newly formed MTs at the centrosome is a complex process that requires the cooperation of many classes of proteins, which need to be recruited to the centrosome (Delgehyr et al., 2005). Among these proteins, some have specific localisation domains that are sufficient for their localisation to the centrosome (*e.g*., AKAP450, pericentrin) (Gillingham and Munro, 2000). Conversely, other proteins are actively transported along MTs (*e.g*., PCM1, centrin, ninein) (Dammermann and Merdes, 2002). Pericentriolar satellites have a pivotal role in this type of transport, by which multi-protein complexes of about 100-nm diameter are transported to the pericentriolar material by dynein–dynactin complexes (Kubo and Tsukita, 2003). Knockdown of the components of pericentriolar satellites (*e.g*., BBS4), or reduced transport of pericentriolar satellites to the centrosome (*e.g*., p50-dynamitin overexpression), have been shown to result in reduced stability of the association of polymerised MTs with the centrosome, and dissociation of the MT network from the centrosome, without altering MT regrowth (Kim et al., 2004; Barenz et al., 2011).

The binding of Cep126 to p150Glued suggests that Cep126 regulates the association of centrosomal cargo proteins to the dynein–dynactin motor complex. Indeed, Cep126 depletion or the transfection with the 1–967 Cep126 deletion mutant did not affect p150Glued localisation, but induced mislocalisation of PCM1 and pericentrin. For this reason, our hypothesis is that Cep126 functions as a cargo receptor for other pericentriolar-satellite components, and is thus crucial for their transport to the centrosome. Of note, the 1–967 Cep126 deletion mutant also interacts with p150Glued. This mutant is localised at the centrosome, but induced a profound alteration of the MT network. For this reason, we propose that this 1–967 Cep126 mutant can compete with endogenous Cep126 and sequester an essential interaction partner. The disruption of Cep126 function also caused alterations in the integrity and positioning of the Golgi apparatus and spindle formation, in line with a function of Cep126 in regulating general MT organisation.

Thus, the impaired cilia assembly observed when Cep126 function is disrupted might be a direct consequence of the impaired pericentriolar-satellite transport to basal bodies, where the cilium is formed. Also, defective MT anchoring might affect cilium formation, as it hampers the localisation of the basal body to the plasma membrane for the direct regulation of ciliogenesis (Kim and Dynlacht, 2013). Thus, the ciliogenesis defect observed here when Cep126 function is impaired might be due to a combination of these two non-exclusive mechanisms.

In conclusion, we propose that Cep126 regulates key centrosomal functions and is consequently required for correct intracellular organisation. Our data are consistent with a model in which Cep126 regulates pericentriolar-satellite recruitment and interacts with p150Glued. This appears to indirectly regulate the MT anchoring to the centrosome, whereby this affects other crucial cellular functions, in turn, such as Golgi integrity, and importantly, cilium formation.

## Materials and Methods

### Cells and antibodies

hTERT-RPE-1 cells were grown in DMEM/F12 (Invitrogen) supplemented with 10% foetal bovine serum, 10 U/ml penicillin and 10 µg/ml streptomycin. COS-7 cells were grown in DMEM (Gibco BRL) supplemented with 10% foetal bovine serum at 37°C in 5% CO_2_.

The anti-KIAA1377/Cep126 antibody (HPA038399), mouse anti-α-tubulin antibodies (clone DM1-A), anti-acetylated-tubulin antibodies (clone 6–11B-1), anti-γ-tubulin antibodies (clone GTU-88), rabbit polyclonal anti-γ-tubulin antibodies (T3559), and rabbit anti-Flag antibodies (F7425) were from Sigma. The rabbit anti-PCM1 (H-262) antibody was from Santa Cruz Biotechnology. The mouse anti-giantin antibody and rabbit anti-Golgin 97 antibody were from Abcam.

### Immunofluorescence

Cells were grown on glass coverslips and fixed in methanol at −20°C for 5 min. The cells were then rinsed in PBS. After blocking in PBS with 1% BSA, the cells were incubated with the primary antibodies diluted in PBS–BSA for 1 h. After washing in PBS, the primary antibodies were detected using anti-Ig secondary antibodies conjugated to Alexa Fluor (Alexa Fluor 488, 568 and 647) (Invitrogen). DNA was stained with 250 ng/ml DAPI for 2 min. Quantification of PCM1 dispersion was carried out as previously described (Kim et al., 2008). Quantification of fluorescence intensities of Cep126, pericentrin, γ-tubulin and p150glued was carried out as previously described (Persico et al., 2010). The cells were mounted in Prolong Gold anti-fade reagent (Invitrogen) and examined on an LSM-710 Carl Zeiss confocal microscope with a 63× NA 1.4 plan apochromat objective. Images were processed using Photoshop CS3.

### Western blotting

hTERT-RPE-1 cells were washed in PBS and lysed on ice in RIPA buffer (100 mM Tris, pH 7.4, 150 mM NaCl, 1% NP40, 0.1% SDS, 0.5% sodium deoxycholate) plus Complete Protease Inhibitor Cocktail (Roche Applied Science). After centrifugation for 20 min at 16,000*g* at 4°C, cleared lysates were obtained. The proteins in the samples were separated using 8% SDS-PAGE gels, followed by their transfer onto nitrocellulose and detection using antibodies.

### Plasmid construction

The KIAA1377 sequence (Source Bioscience) was cloned by PCR with primers containing *Eco*RI (5′-TTAGAATTCACCATGCTGGCGGGGAG-3′) and *Xho*I (5′-ATTCTCGAGCTATCTCTTGTCTCTGCAGC-3′) sites, into p3xFlag. The truncated versions of Cep126 were generated using a PCR-based strategy with primers containing: *Eco*RI (5′-TTAGAATTCACCATGCTGGCGGGGAG-3′) and *Kpn*I (5′-TATGGTACCTCATTCAGCAATTCGTTTTCTTCTC-3′) sites for 1–967; *Eco*RI (5′-TTAGAATTCACCATGCTGGCGGGGAG-3′) and *Kpn*I (5′-TGAGGTACCAACTCCGTCTGAAAATAAAGGCAACTCT-3′) sites for 1–520; *Kpn*I site (5′-GTGGGTACCGGGGGATTAGGAGGATCTGGAGCAGACC-3′) and *Xho*I (5′-ATTCTCGAGCTATCTCTTGTCTCTGCAGC-3′) sites for Δ700; *Kpn*I (5′-GGTACCATGAGTTTTCAAGATGCCTATA-3′) and *Bam*HI (5′- GGATCCTTAAGTCGTAACATTTTCAGAG-3′) sites for 520–655 and cloned into a p3xFlag. All constructs were confirmed by DNA sequencing.

### Cell transfection and siRNAs

Cells were transfected with plasmids using TransIT®-LT1 transfection reagent (Mirus Bio LLC) according to the manufacturer instructions. hTERT-RPE-1 cells and IMCD3 cells (2 × 10^4^ cells seeded per 24-well plate, or 5 × 10^5^ cells seeded per six-well plate) were transfected with 100 nM of a pool of four siRNAs directed against Cep126 (siRNA AK129341:1-GCAGAAAUAUCAAAGACUA; 2-GAAAGACGGAGCAGUAUAU; 3-GAAUCGAGCACGUAAAUAU; 4-GCAACAAAUUGGCGAGCUA; siRNA Cep126: 1-CAAAGUAGCUUCACCGUUA; 2-GCACUUUGGCAUACCGAAA; 3-GCACUGAAUCAUCGGACAA; 4-CGGCAAGAUGCGACAUUAU) as obtained from Dharmacon (ON-TARGETplus Duplex) using Interferin (Polyplus Transfection). After 24 h of transfection, the cells were serum-starved and left for an additional 48 or 72 h before lysis and Western blotting, or methanol fixation and processing for immunofluorescence.

### Live-cell imaging and photobleaching

hTERT-RPE-1 cells were grown on live-cell dishes (0.17-mm-thick coverglass; MatTek) and transiently transfected with Cep126-GFP using Lipofectamine 2000 (Life Technologies, Paisley, UK), according to the manufacturer instructions. Twenty-four hours after transfection, the cells were imaged using a Perkin Elmer UltraVIEW ERS 6FE confocal system spinning disk confocal microscope. Imaging and photobleaching were carried out using a 14 mW argon laser at 488 nm, with a Photokinesis device for region-of-interest photobleaching. Live cells were imaged in DMEM without phenol red, supplemented with 0.1 g/l sodium carbonate and 30 mM HEPES, pH 7.4. Frames were acquired at 1-s intervals. The dynamics of photobleaching were determined by fitting curves to a single exponential using GraphPad 4.02 (Prism). The data in Figure3C were pseudocoloured according to time point using a Temporal-Color Coder ImageJ plugin developed by Kota Miura (EMBL) and available within the Fiji implementation of ImageJ.

### Immunoprecipitation

For purification of Flag-tagged proteins, 1 mg of the whole-cell extract was incubated with 3 µg of the anti-Flag M2 antibody for 16 h at 4°C. Then, 30 µl of washed Protein-G beads (Santa Cruz) was added, and incubated 1 h at 4°C. The beads were then pelleted and washed three times in lysis buffer. The bound protein was eluted by heating the beads to 95°C for 5 min with 1× Laemmli Sample buffer. Inputs represent 1/30th of the extract used for the immunoprecipitation.

### RT-PCR

Total RNA samples from mouse tissues and cell lines were prepared using the RNAeasy Mini kits (Qiagen), followed by cDNA synthesis using Superscript II RT (Invitrogen). Primers: Human KIAA1377 (KIAA1377/CEP126fw: 5′-CCTTTTCCCGTAGACCAACA-3′; KIAA1377/CEP126rev: 5′-CAAGGTTGCCCTCATGTTTT-3′); Mouse KIAA1377(AK12934) (AK12934fw: 5′-TCGGAATCGAGCACGTAAAT-3′; AK12934rev: 5′-GCTGCAGGATCCGTTCTCTA-3′).

### MT regrowth assays

hTERT-RPE-1 cells were transfected with a non-targeting siRNA and the siRNAs against KIAA1377/Cep126. Forty-eight hours later, the cells were treated with 33 µM NZ at 37°C for 3 h. After removing the NZ, the cells were incubated for various times in medium at 37°C, to allow MTs to regrow, as visualised by immunofluorescence microscopy using an anti-acetylated-tubulin antibody.

## Author contribution

R.B., A.L., D.J.S. and A.C. conceived and designed the experiments; R.B., D.W. and A.K.B. performed the experiments; R.B., A.L., D.J.S. and A.C. analysed the data; R.B., D.W., and A.K.B. contributed reagents/materials/analysis tools; R.B., D.J.S. and A.C. wrote the paper; all authors approved the manuscript content and its final version.

## Funding

Funding is acknowledged from the Medical Research Council (grant number G0801848) and the Wellcome Trust for funding to DJS, the Italian Association for Cancer Research (AIRC, Milan, Italy) for financial support to AC (grant number IG6074) and the European Grant Eucilia to AL (HEALT-F2-2007-201804). The Wolfson Bioimaging Facility at the University of Bristol was established through funding from the Medical Research Council Infrastructure Award and the Wolfson Foundation.

## Abbreviations:

hTERT-RPE-1 cells: human telomerase immortalised retinal pigmented epithelial cells
MT: microtubule
NZ: nocodazole.

## Supporting Information

Disclaimer: Supplementary materials have been peer-reviewed but not copyedited.
