# Supplementary figures and images for: Cep126 is required for pericentriolar satellite localisation to the centrosome and for primary cilium formation
Source: Biol Cell. 2014 Jul 9;106(8):254–67. doi: 10.1111/boc.201300087 (PMC4293463; doi:10.1111/boc.201300087)

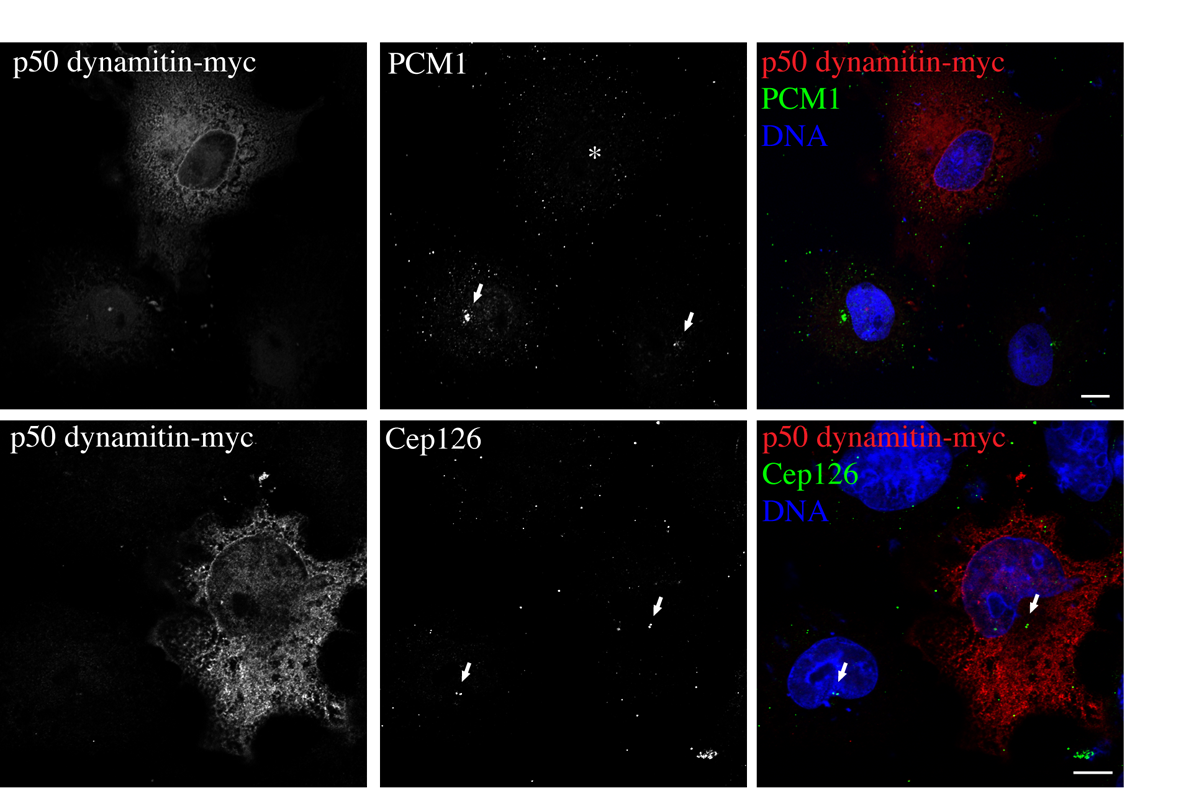

Supplement: Supplementary file 1 — Figure S1 [file boc0106-0254-sd1.tiff]

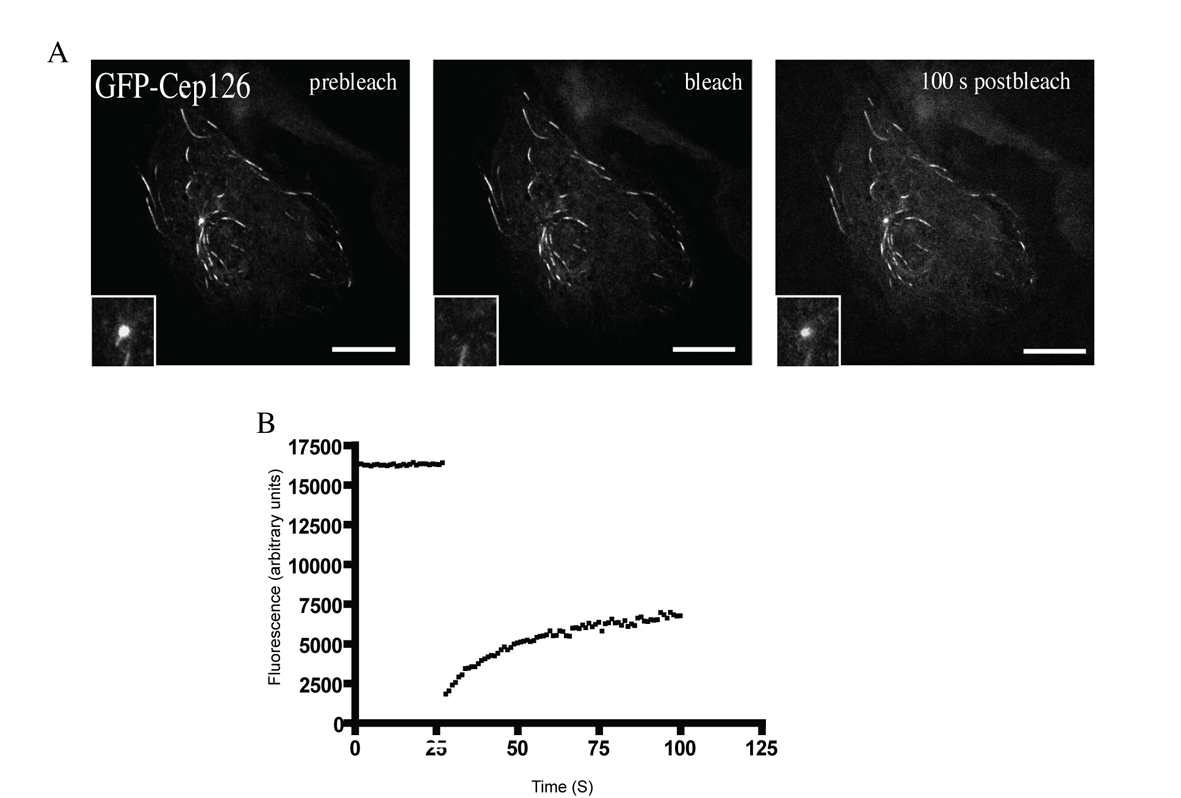

Supplement: Supplementary file 2 — Figure S2 [file boc0106-0254-sd2.tiff]

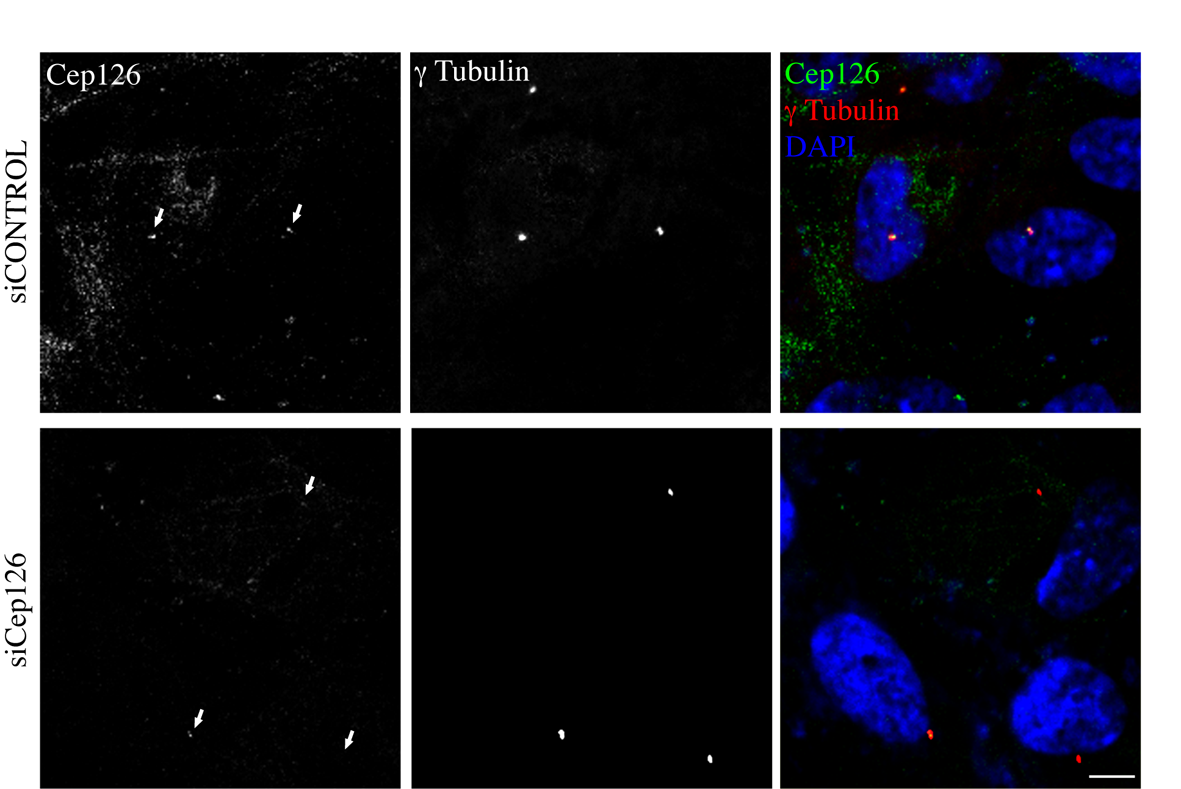

Supplement: Supplementary file 3 — Figure S3 [file boc0106-0254-sd3.tiff]

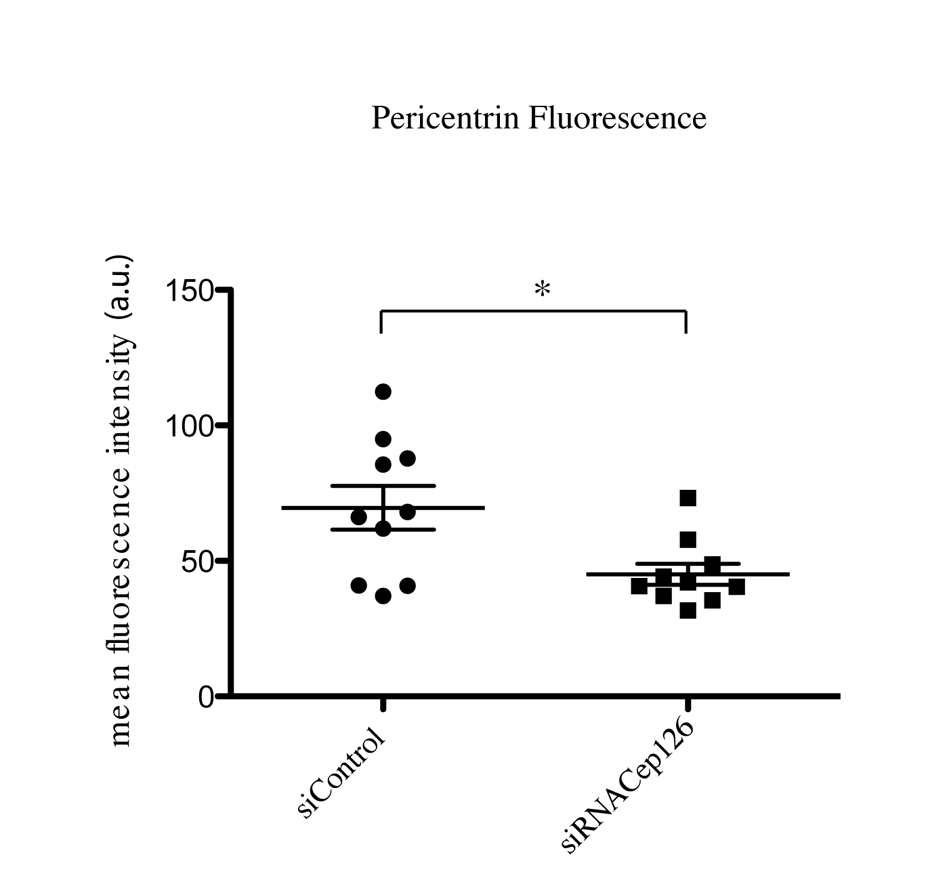

Supplement: Supplementary file 4 — Figure S4 [file boc0106-0254-sd4.tiff]

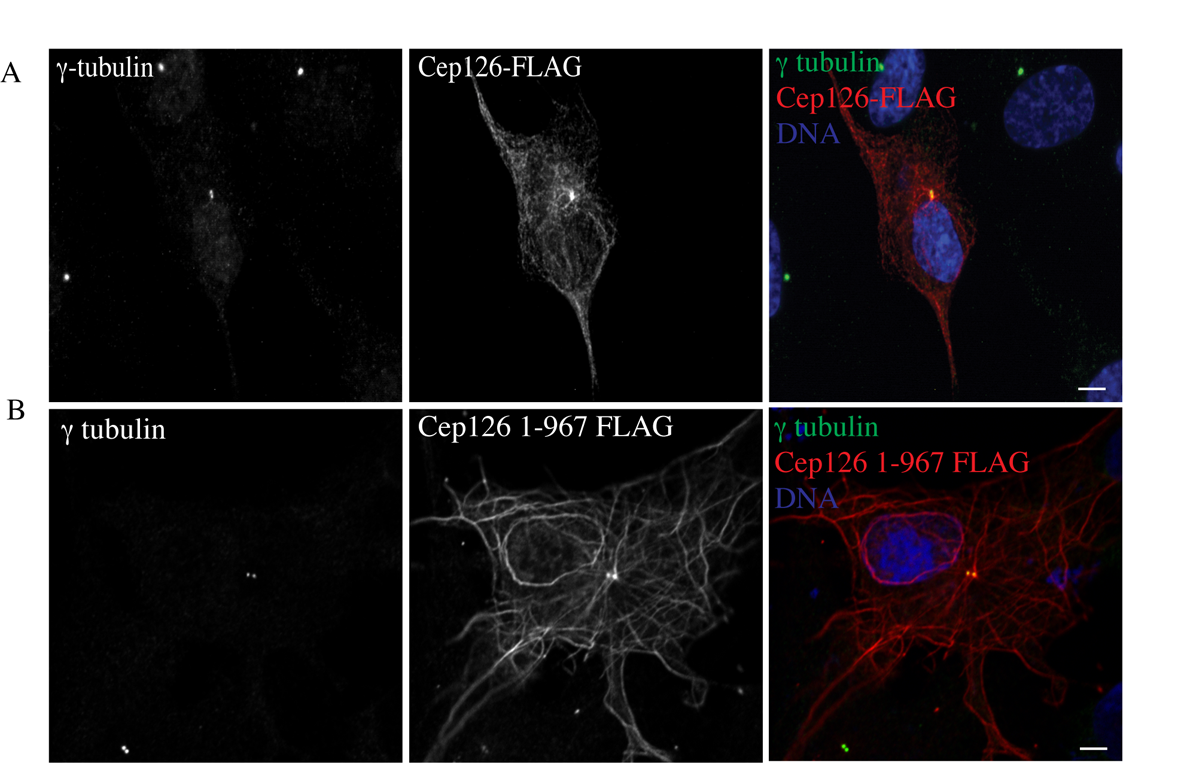

Supplement: Supplementary file 5 — Figure S5 [file boc0106-0254-sd5.tiff]

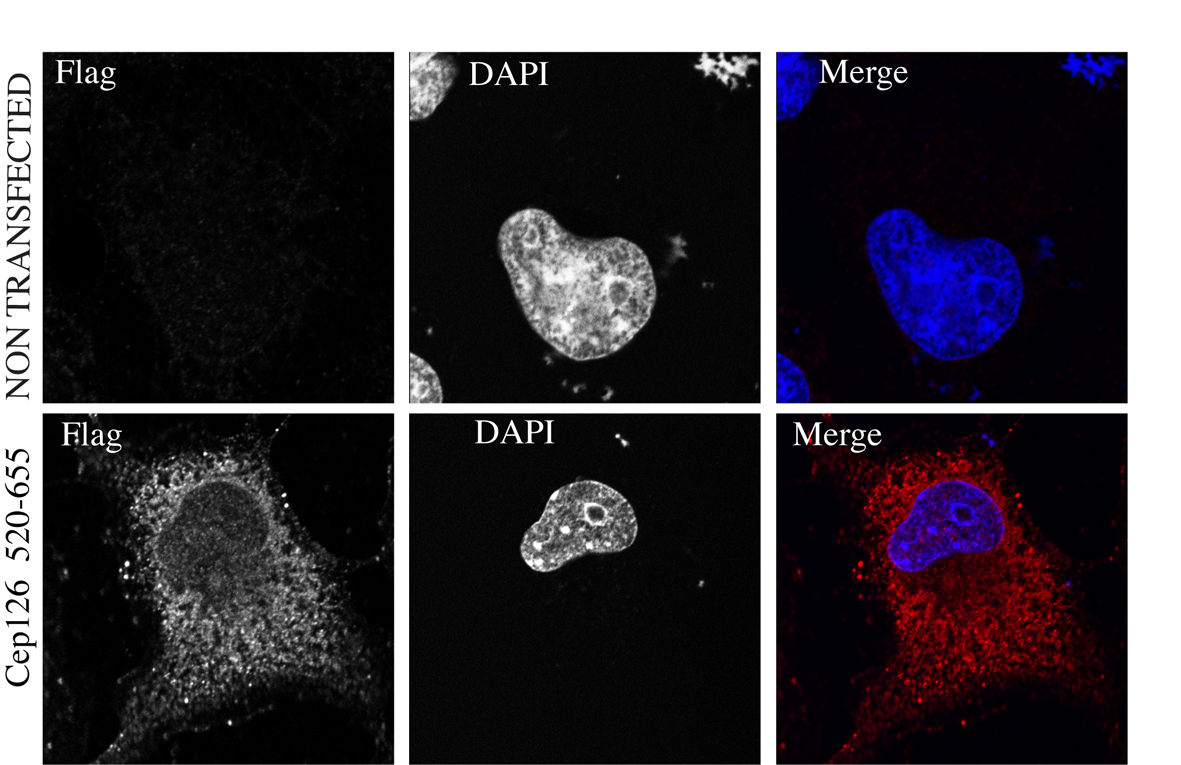

Supplement: Supplementary file 6 — Figure S6 [file boc0106-0254-sd6.tiff]

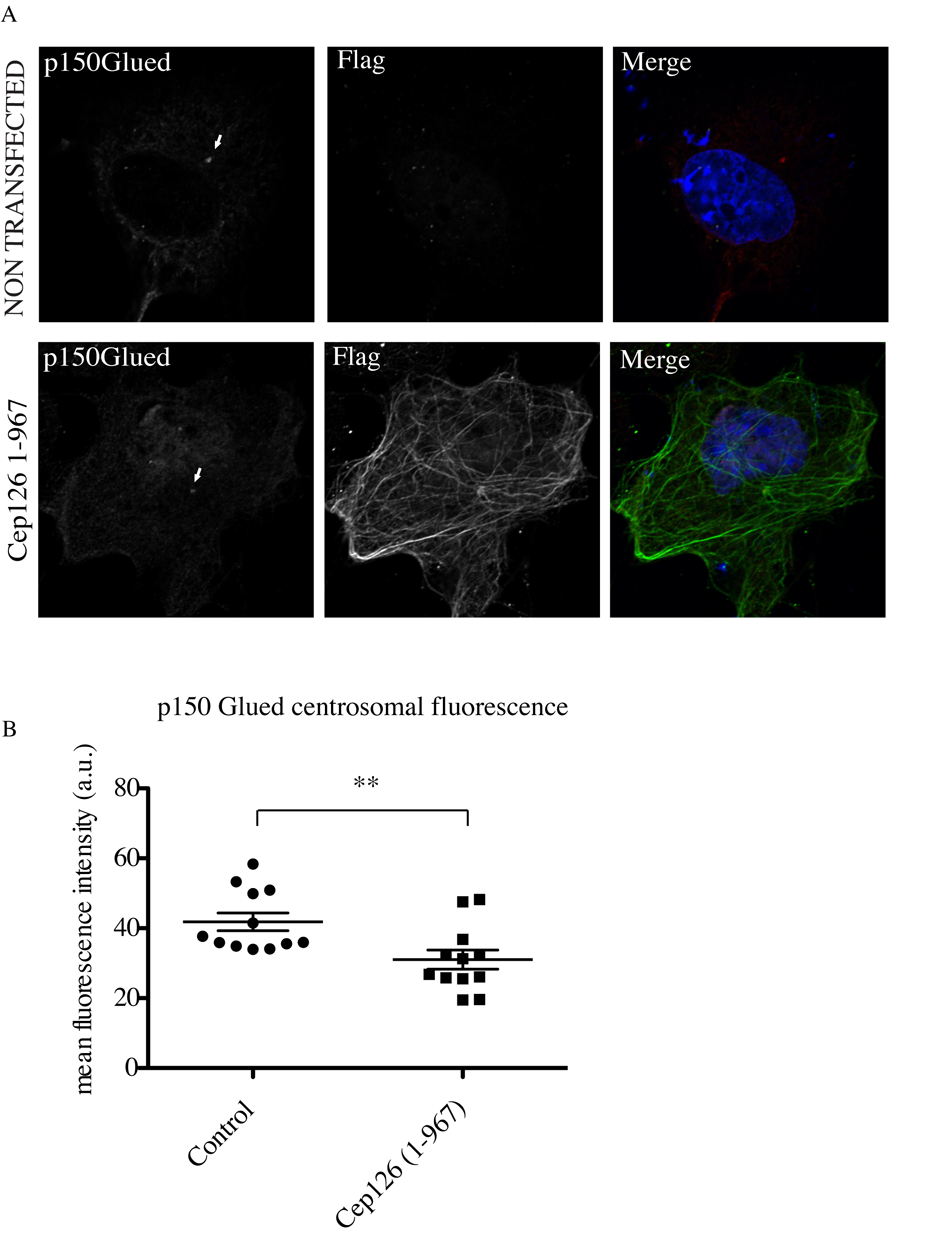

Supplement: Supplementary file 7 — Figure S7 [file boc0106-0254-sd7.tiff]

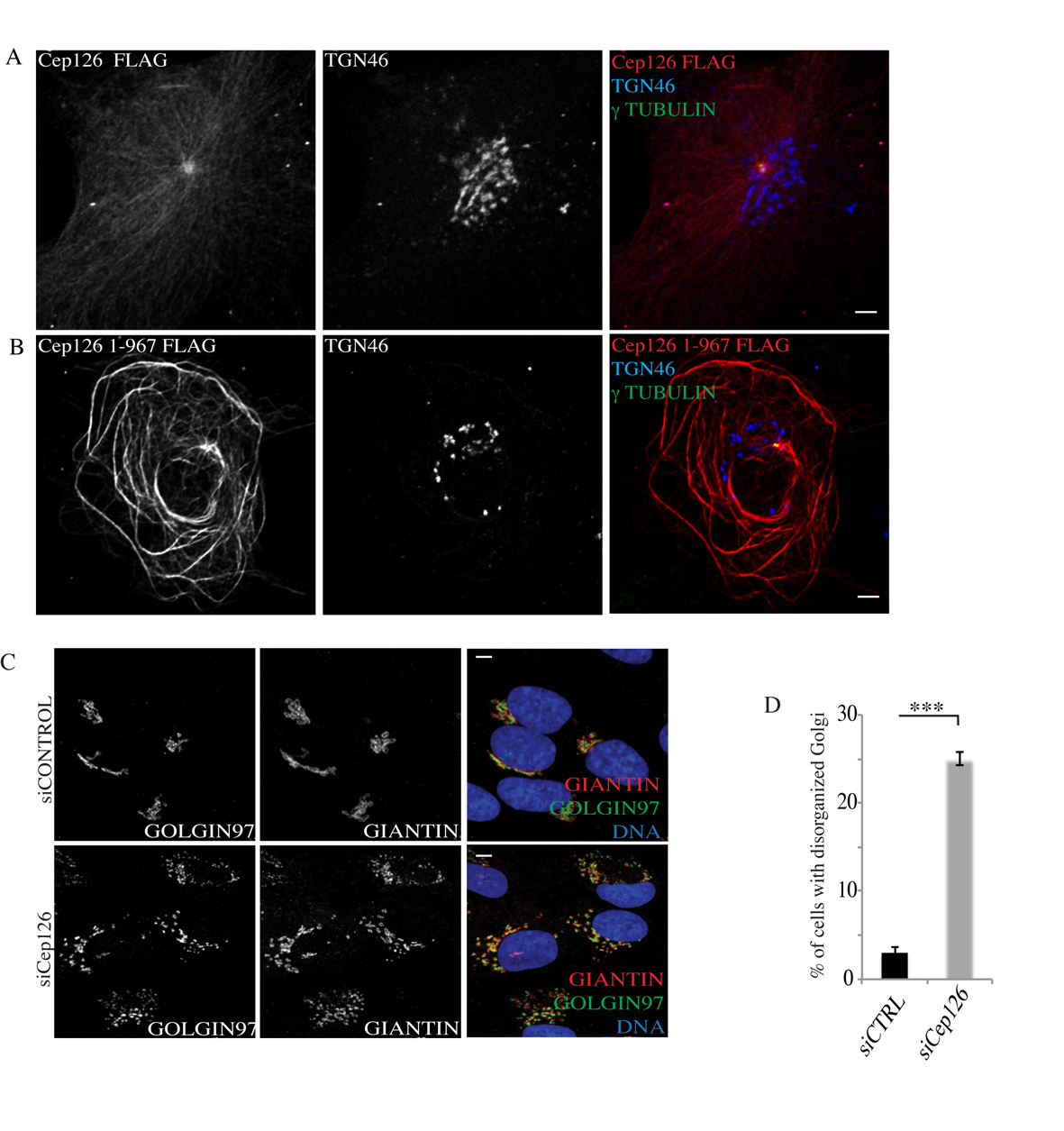

Supplement: Supplementary file 8 — Figure S8 [file boc0106-0254-sd8.tiff]
