# Supplementary material for: Cep126 is required for pericentriolar satellite localisation to the centrosome and for primary cilium formation
Source: Biol Cell. 2014 Jul 9;106(8):254–67. doi: 10.1111/boc.201300087 (PMC4293463; doi:10.1111/boc.201300087)
Supplement: Supplementary file 9 — boc201300087-sup-0009-test.docx [file boc0106-0254-sd9.docx]

**Supplementary movie.** Dynamic localization of Cep126. hTERT-RPE-1 cells were transfected with Cep126-GFP. The dynamics of Cep126 localisation were investigated using live-cell imaging using a confocal microscope.

**Supplementary Figure 1.** hTERT-RPE-1 cells were transfected for 24 hours with the myc-tagged p50dynamitin. The cells were fixed with methanol and stained with antibodies against myc (red), Cep126 (green) or PCM1 (green, arrowheads). p50dynamitin transfection induces PCM1 dispersion (upper panel, asterisk) but does not affect Cep126 localization to the centrosome (bottom panel, arrows). Scale bars: 4 µm.

**Supplementary Figure 2**. hTERT-RPE-1 cells were transfected with Cep126-GFP. (A) Representative images of fluorescence recovery after photobleaching for the dynamics of the centrosomal pool of Cep126-GFP. (B) quantification of the fluorescence recovery of Cep126. Scale bars: 10 µm.

**Supplementary Figure 3.** hTERT-RPE-1 cells were transfected with control siRNA or with siRNA against Cep126 for 48 hours. Cells were fixed and stained with antibodies against  (green) and γ-tubulin (red) to visualise the centrosome. Cep126 depletion induces a reduction of Cep126 staining. Centrosome localization of Cep126 in knock down cells is indicated by an arrow.

**Supplementary Figure 4.** hTERT-RPE-1 cells were transfected with control siRNA or with siRNA against Cep126 for 48 hours. Cells were fixed and stained with antibodies against pericentrin. Cep126 depletion induces a reduction of the pericentrin fluorescence to the centrosome. The analysis was performed as indicated in material and methods.

**Supplementary Figure 5.** The Cep126 truncation mutant localises to the centrosome. hTERT-RPE-1 cells were transfected with full-length Cep126-Flag (A) and with 1-967 Cep126-Flag (B), fixed with methanol, and stained with anti-γ-tubulin antibodies (red) and with anti-Flag antibodies (green).

**Supplementary Figure 6.** hTERT-RPE-1 cells were transfected with 520-655 Cep126 deletion mutant, fixed with methanol and stained with an anti Flag antibody and with DAPI.

**Supplementary Figure 7.** Effect of 1-967 Cep126 mutant on p150Glued localization. hTERT-RPE-1 cells were transfected with 1-967 Cep126-Flag to induce the formation of MT bundles. (A) The cells were fixed with methanol and stained with antibodies against Flag (green) and p150Glued (red). p150Glued fluorescence at the centrosome was reduced after 1-967 Cep126 transfection. (B) Quantification of p150 fluorescence at the centrosome. p150Glued fluorescence was measured as indicated in materials and methods.

**Supplementary Figure 8.** hTERT-RPE-1 cells transfected with full-length Cep126-Flag (A) or with 1-967 Cep126 **(**B**)** for 24 hours, fixed and stained with antibodies against Flag (red) γ tubulin (green) and TGN 46 (blue). 1-967 Cep126 truncation mutant affects Golgi complex morphology. (C, D) hTERT-RPE-1 cells were transfected with control siRNA or with siRNA against Cep126 for 48 hours. (C) RPE-1 cells stained with giantin (red) and golgin-97 (green) antibodies as markers of the Golgi complex. (D) The graph represents the percentage of cells showing a disorganized Golgi complex. Cep126 depletion affects Golgi complex morphology. *** indicates p< 0.0001, using t test. Data in (D) are means ±s.d. of three independent experiments; more than 100 cells where counted per experimental condition.
